# Complications secondary to cosmetic artificial iris anterior chamber implants: a case report

**DOI:** 10.1186/s12886-015-0084-1

**Published:** 2015-08-08

**Authors:** Yusrah Shweikh, Sally Ameen, Ali Mearza

**Affiliations:** Moorfields Eye Hospital, London, UK; Imperial College Healthcare NHS Trust, Charing Cross Hospital, Fulham Palace Road, W6 8RF London, UK

**Keywords:** Artificial iris implants, NewColorIris, Adverse effects

## Abstract

**Background:**

Artificial iris anterior chamber implants were originally developed for therapeutic purposes but have been used recently for the cosmetic alteration of eye colour. There is a growing body of evidence surrounding their associated risks. We report a case presenting with complications secondary to bilateral NewColorIris® implants, including the first report of pressure-induced stromal keratopathy in this context.

**Case presentation:**

A thirty-eight year old South American man presented as an emergency in the UK with best corrected visual acuities of 1/60 OD and 6/18 OS, bilateral corneal decompensation, lens opacities and raised intraocular pressures 4 years following bilateral NewColorIris® implantation in Panama. Anterior segment optical coherence tomography demonstrated the direct apposition of the implant with the iris and iridocorneal angle, together with pressure-induced stromal keratopathy with a fluid interface between the corneal stroma and previous laser-assisted in situ keratomileusis flaps. We describe the successful combined medical and surgical management in this case to yield a final visual acuity 6/12 in both eyes.

**Conclusion:**

Artificial iris anterior chamber implants are associated with sight-threatening complications that can present years after their implantation. We caution against their use for the cosmetic alteration of eye colour.

## Background

Silicone anterior chamber iris implants were first developed by Kahn in 2004 [1]. Their original intended use when first presented at the American Academy of Ophthalmology meeting was for the treatment of oculocutaneous albinism, as well as to improve the iris appearance of patients with hereditary defects such as iris colobomas or traumatic iris damage. However, they soon became promoted as a cosmetic implant for people wishing to change their iris colour.

We describe a case of bilateral secondary glaucoma and severe corneal decompensation four years following the implantation of NewColorIris® devices in Panama. This case emphasises the dangers of a procedure advertised as a safe alternative for cosmetic coloured contact lenses and highlights the fact that sequelae can develop years after the original surgery.

## Case presentation

**Fig. 1:**
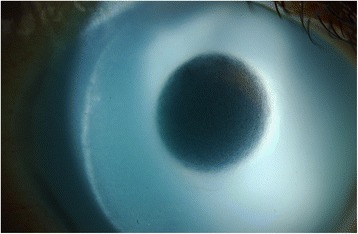
Anterior chamber slit lamp image. The implant is seen covering the entire iris, with signs of corneal decompensation

A thirty eight year old South American man presented to eye casualty with a four-week history of bilateral progressive visual loss. His past ophthalmic history includes laser-assisted in situ keratomileusis (LASIK) five years previously and cosmetic anterior chamber iris implants (NewColorIris®) four years prior to presentation. These operations were performed in Panama. At presentation, best-corrected visual acuity (BCVA) with glasses was 1/60 (2/60 with pinhole) right eye (OD) and 6/18 (6/9 with pinhole) left eye (OS). Anterior segment examination revealed the presence of blue angle-supported iris implants and bilateral corneal decompensation with stromal oedema and endothelial pigment deposition (Fig. 1). Intraocular pressure (IOP) at presentation was 40 mmHg in both eyes and on gonioscopy all angle structures were obscured by the implants. There were no peripheral iridotomies seen. In addition, bilateral lens opacities were present (left eye lens opacities worse than right).

**Fig. 2:**
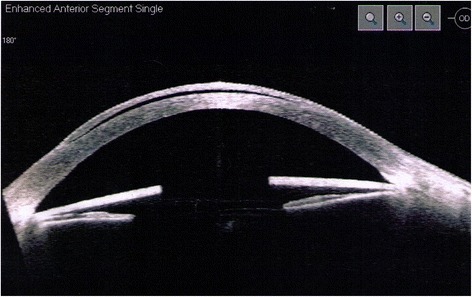
Optical coherence tomography of the right eye showing the position of the iris implant in direct contact with underlying iris tissue and obstructing the iridocorneal angle. Fluid is demonstrated in the interface between the LASIK flap and underlying corneal stroma

Anterior segment optical coherence tomography confirmed that the iris implants were in direct contact with both the cornea and iris. Imaging also demonstrated fluid in the interface between the LASIK flaps and underlying corneal stroma (Fig. 2).

The patient was immediately commenced on two hourly G. dexamethasone 0.1 % preservative-free drops and G. Ganfort® (bimatoprost 0.3 mg/mL and timolol 5.0 mg/mL) once a day to both eyes. He was reviewed three days later, when his BCVA had reduced to counting fingers (CF) at 1 m OD and 6/36 (6/12 with pinhole) OS. IOP was recorded at 18 mmHg in both eyes.

After obtaining fully informed consent, the patient underwent removal of the anterior chamber implants under general anaesthesia. The implants were removed through a temporal corneal tunnel. The anterior chamber was first formed with a cohesive viscoelastic (Healon®). A 2.75 mm corneal incision was then made. Two sphincterotomies were performed to facilitate the folding of the implants. The implants were mobilised, folded and removed via the wound. The wound was then secured with 10/0 Nylon sutures after the removal of the viscoelastic.

One week post-operatively, the BCVA remained at CF at 1 m in both eyes due to persistent corneal decompensation. The IOP in the left eye began to rise despite good compliance with the drops (from 18 mmHg to 28 mmHg). G. apraclonodine 0.5 % was commenced three times per day in the left eye.

The corneal decompensation unfortunately did not improve. With consent, the patient underwent right Descemet stripping endothelial keratoplasty (DSEK) under general anaesthesia one month after his initial presentation. He subsequently underwent left phacoemulsification with intraocular lens implantation combined with DSEK under general anaesthesia nine months after his original presentation. Both operations were uneventful.

**Fig. 3:**
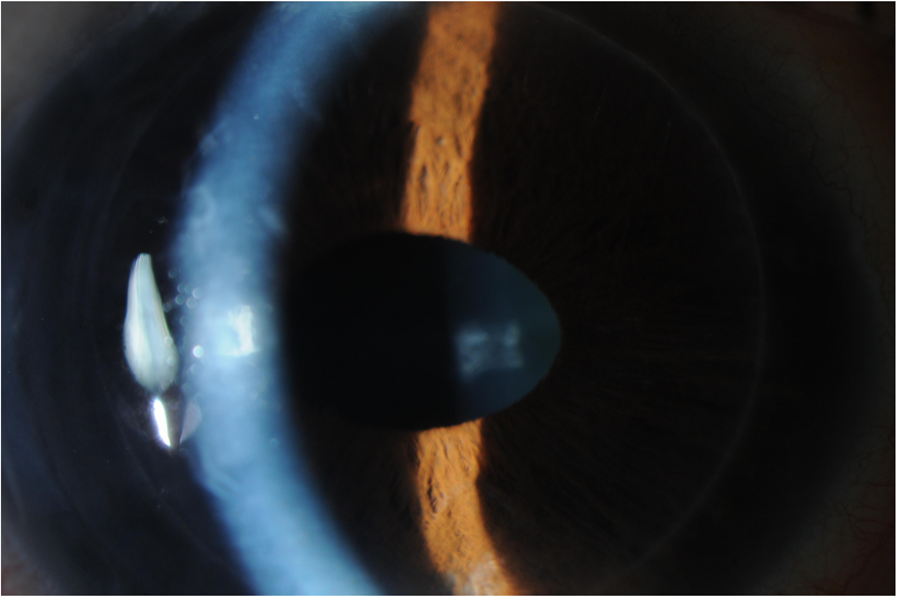
Post-operative view of the right eye. The anterior chamber is deep with a clear cornea post-DSEK

At final follow up six months after his left eye surgery, the patient’s BCVA was 6/12 in the right eye and 6/12 in the left eye. Both anterior chambers were deep and quiet, with clear, well-centred grafts (Fig. 3). The IOP was 11 mmHg OD and 14 mmHg OS. On gonioscopy the angle was open on 360° inspection in the right eye. In the left eye there were 270° of broad peripheral anterior synechiae (PAS). Fundus examination showed a healthy optic disc in the right eye with a cup:disc (C:D) ratio of 0.2 and healthy neuroretinal rim. The left eye however did show evidence of neural rim loss mainly superiorly with a C:D ratio of 0.7. The patient was being maintained on G. dexamethasone 0.1 % drops once a day to each eye and G. timolol 0.5 % twice a day to the left eye.

## Conclusions

The cosmetic use of intraocular implants is a relatively new phenomenon in the UK [2]. Although there are no robust studies assessing the long-term safety and efficacy of these implants, there have been a growing number of case reports and case series highlighting the dangers of artificial iris anterior chamber implants [2–10]. These include the permanent structural damage of the corneal endothelium causing corneal decompensation [8], complete stromal atrophy of iris adjacent to NewColorIris® implants [9], uveitis, central retinal vein occlusion, uncontrolled rise in IOP and secondary glaucoma [8]. The biggest case series to date was published by Hoguet, et al [7], who report on 14 eyes, 7 (50 %) of which had a raised IOP at presentation. The majority could not be managed with topical treatment alone and required surgical intervention with either trabeculectomy or aqueous drainage devices. A review by Sikder, et al found that 81.25 % of phakic eyes developed corneal oedema following NewColorIris® implantation [10].

The NewColorIris® is a single-piece silicone implant. It is single-sized with a diameter of 15 mm, a central pupillary aperture of 4.5 mm and 0.16 mm thickness [1]. The implant is folded and inserted through a 2.8 mm corneal incision. It is unfolded in the anterior chamber and positioned directly above the iris. The implant has six foot processes to secure its position and in theory only partially occlude the angle [11]. The implant is circular and it is therefore anticipated that it is not supported in a uniform way within the anterior chamber given the anatomical asymmetry between vertical and horizontal meridians [10]. Anderson, et al have demonstrated vaulting of implants in the anterior chamber [5] and there are also concerns over changing implant position over time [10].

Angle supported anterior chamber implants have been in use since the 1950’s for the correction of aphakia [12]. They are well known to cause rises in IOP, reportedly in up to 75 % of patients [10]. The direct implant contact with the iris results in continuous mechanical damage and pigment dispersion. The liberated pigment consequently occludes the trabecular meshwork and leads to rises in IOP. Castanera et al also reported surface irregularities of the NewColorIris® implants on electron microscopy. These irregularities can further exacerbate the dispersion of iris pigment and may contribute to endothelial cell damage [13]. The implant can also cause pupillary block if no prophylactic peripheral iridotomy is performed at the time of implantation. In our case, we hypothesise that the rise in IOP was due to a combination of pigment dispersion and mechanical trabecular meshwork damage resulting from the direct apposition of the implant with iridocorneal angle structures. These mechanisms have also been documented by other authors [2–5,13]. This is likely to have induced the formation of PAS in the left eye, contributing to continued rises in IOP despite implant removal. The pigment dispersion observed in both eyes improved post-operatively.

In all published case reports, implants were surgically explanted. Sikder and co-authors emphasise the importance of minimising intraoperative trauma to the iris, lens and corneal endothelium. They describe a surgical technique for NewColorIris® implant removal that involves cutting the implant into three equal segments and removing each through a 2.75 mm temporal clear corneal incision [14]. Despite careful explantation, the damage induced by artificial iris anterior chamber implants can be permanent. Our patient developed secondary corneal endothelial cell loss, glaucoma and cataracts as a result of NewColorIris® implants, as well as pressure-induced stromal keratopathy (PISK) (Fig. 2). The latter complication has not been described previously in this context and to the best of our knowledge is unique in our patient. PISK is a rare condition affecting eyes that have undergone lamellar corneal refractive surgery [15]. In this case, the patient underwent LASIK one year prior to NewColorIris® implantation and PISK occurred secondary to endothelial cell failure and high IOP. An important consideration is the purported underestimation of IOP by applanation tonometry in the prescence of interface fluid [16]. A complete resolution of interface fluid was observed in both eyes following topical IOP-lowering treatment, iris implant removal and subsequent combined phacoemulsification with intraocular lens insertion and DSEK.

This case adds to the literature by highlighting the long-term sight-threatening sequelae of NewColorIris® implants. There has been increasing interest in recent years over the cosmetic use of these implants, with the procedure being advertised by informal websites with a lack of medical review or published evidence to support their safety [17]. We caution against their use for the cosmetic alteration of iris colour.

## Consent

Written informed consent was obtained from the patient for publication of this Case report and any accompanying images. A copy of the written consent is available for review by the Editor of this journal.

## Abbreviations

LASIK: Laser-assisted in situ keratomileusis
BCVA: Best-corrected visual acuity
IOP: Intraocular pressure
CF: Counting fingers
DSEK: Descemet stripping endothelial keratoplasty
PAS: Peripheral anterior synechiae
C:D: Cup:disc ratio
PISK: Pressure-induced stromal keratopathy
